# Clinical Efficacy and Safety of Reduced-Dose Prasugrel After Percutaneous Coronary Intervention for Taiwanese Patients with Acute Coronary Syndromes

**DOI:** 10.3390/jcm13237221

**Published:** 2024-11-27

**Authors:** Hsun-Hao Chang, Chi-Feng Hung, Yen-Ju Chen, Ching-Chang Fang

**Affiliations:** 1 Department of Cardiology, Tainan Municipal Hospital (Managed by Show Chwan Medical Care Corporation), Tainan 701033, Taiwan; hsunhaw0520@gmail.com; 2 Pharmaceutical Biotechnology, Fu-Jen Catholic University, New Taipei City 242062, Taiwan; 054317@gmail.com; 3 School of Medicine, Fu-Jen Catholic University, New Taipei City 242062, Taiwan; 4 Research Assistant Center, Tainan Municipal Hospital (Managed by Show Chwan Medical Care Corporation), Tainan 701033, Taiwan; 5 Department of Food Nutrition, Chung Hwa University of Medical Technology, Tainan 717302, Taiwan

**Keywords:** major adverse cardiovascular events, bleeding, percutaneous coronary intervention, acute coronary syndromes, prasugrel

## Abstract

**Background/Objectives**: The efficacy and safety of reduced-dose prasugrel (loading dose/maintenance dose: 20/3.75 mg) in preventing major adverse cardiovascular events (MACEs) among patients with acute coronary syndrome (ACS) undergoing percutaneous coronary intervention (PCI) have been well-estab-lished. However, long-term real-world data for this population remains limited in Taiwan. **Methods**: This study enrolled 226 Taiwanese ACS patients (with 448 lesions) who received reduced-dose pra-sugrel after PCI and completed one year of follow-up. **Results**: The primary efficacy outcome was the in-cidence of MACEs. After one year, the MACE rate was 7.1% (16/226). A comparative analysis of MACEs was conducted across subgroups stratified by age (<75 vs. ≥75 years), body mass index (<25 vs. ≥25 kg/m^2^), body weight (<60 vs. ≥60 kg), and estimated glomerular filtration rate (<60 vs. ≥60 mL/min/1.73 m^2^). Patients with impaired renal function had a 4.03-fold higher risk (95% con-fidence interval = 1.37–11.90, *p* = 0.01) of MACEs than those with optimal renal function. The primary safety endpoint was major bleeding events (Bleeding Academic Research Consortium types 3 or 5), which occurred in 0.8% (2/226) of patients, all gastrointestinal. The secondary end-point was net adverse clinical events (NACEs), a composite of MACEs and major bleeding, with an observed rate of 8.0% (18/226). **Conclusions**: Reduced-dose prasugrel demonstrated both safety and efficacy in Taiwanese ACS patients undergoing PCI.

## 1. Introduction

Acute coronary syndromes (ACSs) encompass a spectrum of life-threatening conditions, such as ST-elevation myocardial infarction (STEMI), non-ST-elevation myocardial infarction (NSTEMI), and unstable angina. Each year, over seven million individuals worldwide are diagnosed with these conditions [1]. In Taiwan, coronary artery disease (CAD) is responsible for over 17,000 deaths annually [2].

Percutaneous coronary intervention (PCI) combined with optimal pharmacological management form the foundation of therapy for ACS. Among the pharmacological strategies, initiating dual antiplatelet therapy (DAPT), comprising aspirin and a P2Y12 inhibitor, is essential. Numerous studies have confirmed the efficacy of DAPT in reducing severe complications, such as acute, subacute, and late stent thrombosis (ST), and preventing recurrent ischemic cardiovascular events post-PCI in individuals with ACS [3,4,5]. Compared to clopidogrel, more potent P2Y12 inhibitors, including prasugrel and ticagrelor, have a related reduction in major adverse cardiovascular events (MACEs) among ACS patients [6]. Especially, the TRITON-TIMI 38 trial highlighted that prasugrel, administered as a 60 mg loading dose followed by a 10 mg daily maintenance dose, significantly reduced the occurrence of ischemic events, such as ST, in comparison to clopidogrel for ACS patients undergoing PCI. Nonetheless, the trial also revealed an elevated risk of bleeding without an apparent benefit from prasugrel in certain patient demographics: those aged 75 years or older, individuals weighing less than 60 kg, and patients with a history of transient ischemic attack (TIA) or ischemic stroke [7]. In light of these observations, a Phase II clinical trial employing a randomized, double-blind, dose-optimization strategy for prasugrel was conducted among Japanese patients undergoing PCI. The study demonstrated that a lower prasugrel regimen, comprising a 20 mg loading dose (LD) followed by a 3.75 mg daily maintenance dose (MD), provided a strong antiplatelet effect while simultaneously reducing the risk of bleeding events [8]. Additionally, the RENAMI and BleeMACS registries showed that prasugrel significantly reduced the incidence of major adverse cardiovascular events (MACEs) (2.6% vs. 5.2%, *p* = 0.007) and net adverse clinical events (NACEs) (4.2% vs. 7.6%, *p* = 0.002) at one year in ACS patients undergoing PCI compared to clopidogrel. These findings suggest that prasugrel offers a more favorable balance between preventing ischemic events and the risk of recurrent bleeding [9].

A reduced dosage of prasugrel was introduced in Taiwan at the end of 2018. Tainan Municipal Hospital, managed by Show Chwan Medical Care Corporation, began implementing this reduced dosage protocol for prasugrel in May 2019. This manuscript aims to elucidate the outcomes of a one-year follow-up study evaluating the efficacy and safety of the reduced prasugrel dose in ACS patients undergoing PCI, as one-year DAPT is a Class I recommendation globally for ACS patients undergoing PCI [10].

## 2. Materials and Methods

### 2.1. Subjects

**Figure 1:**
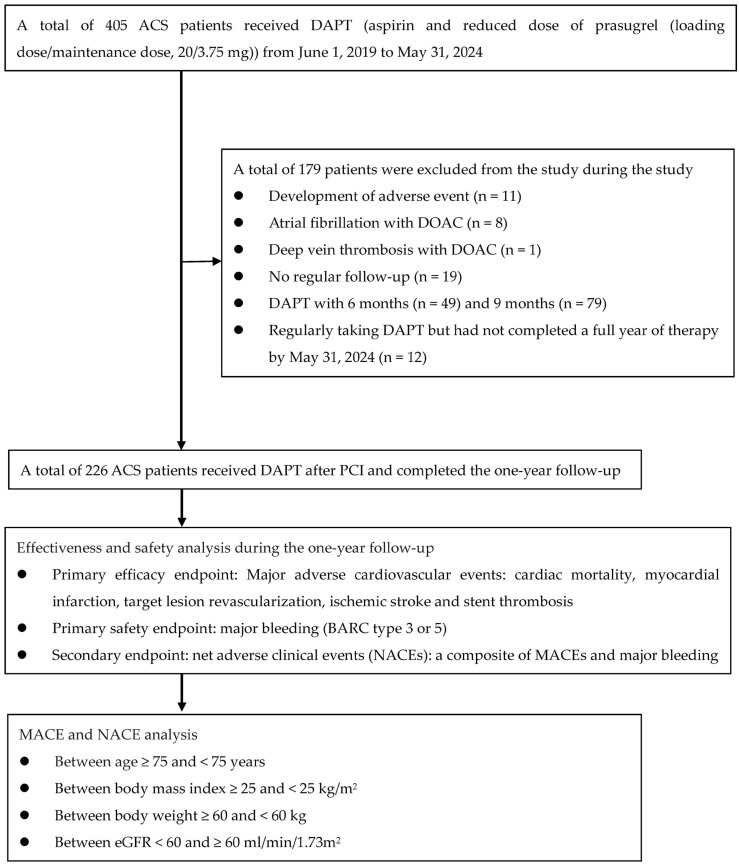
Study flow diagram. ACS, acute coronary syndrome; DAPT, dual antiplatelet therapy; DOAC, direct oral anticoagulant; PCI, percutaneous coronary intervention; BARC, Bleeding Academic Research Consortium; MACEs, major adverse cardiovascular events; NACEs, net adverse clinical events; eGFR, estimated glomerular filtration rate.

This retrospective cohort study aimed to assess the efficacy and safety of a reduced-dose prasugrel regimen (LD/MD, 20 mg/3.75 mg) in patients aged 20 years or older diagnosed with ACS requiring PCI at Tainan Municipal Hospital, managed by Show Chwan Medical Care Corporation, in Tainan, Taiwan. This study was approved by the Institutional Review Board of Show Chwan Memorial Hospital (IRB no: 1120210) and the duration spanned from 1 June 2019 to 31 May 2024 (Figure 1), with a one-year observational period following the initiation of prasugrel treatment, regardless of the patients’ adherence to or discontinuation of the therapy. Previous studies have demonstrated that prasugrel may not provide a net clinical benefit for individuals aged 75 years or older or for those weighing less than 60 kg [7]. Additionally, the impact of the body mass index (BMI) on the efficacy of P2Y12 inhibitors in patients with ACS has been well established [11]. Furthermore, a strong association has been observed between a reduced estimated glomerular filtration rate (eGFR) and an increased risk of major adverse cardiovascular events (MACEs) [12]. A predefined substudy was conducted to compare the outcomes based on age (<75 vs. ≥75 years), body mass index (BMI < 25 vs. ≥25 kg/m^2^), body weight (<60 vs. ≥60 kg), and estimated glomerular filtration rate (eGFR < 60 vs. ≥60 mL/min/1.73 m^2^). The key exclusion criteria initially included an anticipated life expectancy of less than one year, a history of intracranial hemorrhage (ICH), and conditions that increase the risk of bleeding, such as thrombocytopenia purpura, pancytopenia, and hemophilia.

### 2.2. Drug Therapy/Intervention

Prasugrel was prescribed at the discretion of the attending physicians. The LD of prasugrel (20 mg) was administered orally *on the first day of treatment. Thereafter, the MD of prasugrel (3.75 mg) was given orally once daily, starting the day after the LD, to be taken after breakfast. Prasugrel was administered as part of the DAPT alongside aspirin (100 mg/day), following an initial LD of 300 mg.*

### 2.3. Study Variables

The study variables at one year included the patients’ baseline clinical characteristics, initial coronary angiography findings, the administration status of prasugrel, the use of concomitant medications (e.g., angiotensin-converting enzyme inhibitors (ACEIs), angiotensin II receptor blockers (ARBs), sacubitril/valsartan (Entresto), β-blockers and statins) before study inclusion, cardiovascular events, and adverse events (AEs), including bleeding [13].

### 2.4. Definition of Clinical Endpoints

Clinical assessments were conducted during hospitalization, with follow-up appointments scheduled at one-year intervals following PCI [13]. The primary efficacy endpoint was the incidence of MACEs, which included cardiac mortality, non-fatal myocardial infarction (MI), the need for target lesion revascularization (TLR), non-fatal ischemic stroke, and stent thrombosis (ST). Cardiac mortality was defined as death resulting from cardiac causes such as MI, heart failure, or fatal arrhythmias. In cases of unwitnessed or unknown deaths, it was essential to classify them definitively as cardiac death, even among patients with potentially life-threatening conditions such as malignancy or infection. Unless a definite non-cardiac cause was established, the cause of death was considered cardiac-related. MI was defined according to the universal definition, requiring clinical or electrocardiographic evidence of myocardial ischemia accompanied by elevated cardiac troponin levels exceeding the upper limit of normal [14]. Target-vessel-related MI was defined as MI attributable to the target vessel or cases where it was indeterminate whether it was related to non-target vessels. TLR was defined as any repeated PCI performed on the target lesion or surgery on the target vessel due to restenosis or other complications. Stroke was defined as an episode of neurological dysfunction caused by focal cerebral infarction, confirmed by brain imaging with computed tomography (CT) or magnetic resonance imaging (MRI) [15]. ST was categorized into acute (within 24 h), subacute (within 30 days), and late (30 days to 12 months) occurrences, following the definitions set by the Academic Research Consortium (ARC) [16]. The primary safety endpoint was the incidence of bleeding events, with major bleeding defined as Bleeding Academic Research Consortium (BARC) type 3 or 5 bleeds [5,17]. The secondary endpoint was the incidence of NACEs, a composite of MACEs and major bleeding endpoints [18]. The observation period was set to one year as the maximal period for calculating the cumulative incidence.

### 2.5. Statistical Analysis

This study employed standard statistical methods to analyze the clinical characteristics and outcomes. Categorical variables were presented as proportions, while continuous variables were expressed as the mean ± standard deviation. As predictors of MACEs and TLR at 12 months (refer to Supplementary Tables S1 and S2), although eGFR was the key factor for MACEs or TLR, considering that gender, age, BMI/weight, hypertension, hyperlipidemia, diabetes mellitus, and tobacco smoking are commonly included as control variables in the literature, we also incorporated these factors in the subsequent multivariable analysis. A multivariate Cox proportional hazards regression model examined the relationship between risk factors and outcomes. This model provided estimates of the hazard ratio (HR) and 95% confidence interval (CI) for the primary and secondary composite outcomes. The Kaplan–Meier analysis calculated the observed event rate at one year and the cumulative incidence of event-free survival. For the survival analysis, the Kaplan–Meier (KM) method considers only a single event, with other scenarios treated as censored, to present survival rates at different time points. Given that patients may face other competing risks, this study incorporated the concepts of a cause-specific hazard function and sub-distribution hazard function to assess the cumulative incidence of MACEs and the mortality risk within one year (Supplementary Figure S1). Differences between the two groups were assessed using the log-rank test. Analyses were conducted using SPSS version 22.0 (IBM Corp., IBM SPSS Statistics for Windows, Armonk, NY, USA) and R (v4.4.2, R Core Team 2024). A *p*-value of less than 0.05 was considered statistically significant.

## 3. Results

### 3.1. Baseline, Lesion, and Procedural Characteristics of the Study Population

A cohort of 405 consecutive patients with ACS requiring PCI were enrolled in this study. During the study period, 179 patients were excluded for various reasons: 11 patients experienced adverse events following DAPT administration and did not receive reduced-dose prasugrel after these adverse events occurred. The adverse events included three cases of dyspnea, four cases of ecchymosis, one case of hematuria, two cases of allergy, and one case of nasal bleeding. Additionally, eight with atrial fibrillation and one with deep vein thrombosis were treated with prasugrel and direct oral anticoagulants. Furthermore, 21 patients were excluded due to miscellaneous reasons. Given that the primary aim of our study was to analyze the effectiveness and safety of DAPT within one year of its use, we excluded patients who had received DAPT for only 6 months (49 patients) and 9 months (79 patients) following PCI. Twelve patients who consistently adhered to DAPT but had not completed one year of therapy by the 31 May 2024 cut-off. Consequently, the final safety and efficacy analysis was performed on a subset of 226 patients, as shown in Figure 1.

The baseline clinical characteristics of the study cohort are detailed in Table 1. The analysis included a total of 226 patients. The mean age of the participants was 66.1 ± 11.1 years, with a predominant male representation (182/226, 80.5%). Overweight and obesity were common, with nearly 60% (129/226, 57.1%) having a BMI greater than 25 kg/m^2^. The average body weight was 70 ± 11.7 kg, with 75% (170/226) of patients weighing over 60 kg. The patients’ past medical histories revealed incidences of MI in 11.1%, non-fatal ischemic stroke in 10.6%, atrial fibrillation in 3.5%, and ESRD in 7.1%. According to the definition of the Academic Research Consortium for High Bleeding Risk (ARC-HBR) criteria for patients undergoing PCI [19,20], 94 (41.6%) patients were in the HBR group. We categorized the ACS patients by PCI complexity, defined as meeting at least one of the following criteria: treatment of three vessels, implantation of three or more stents, bifurcation with two stents, a total stent length of 60 mm or more, PCI of the left main coronary artery, or targeting a chronic total occlusion (CTO) [21]. Table 1 shows the prevalence of complex PCI within the whole population. Common concomitant diseases included hypertension (51.8%), hyperlipidemia (72.6%), diabetes mellitus (38.9%), and chronic kidney disease (CKD) stage 3, 4, 5, and ESRD (34.1%). Additionally, 26.1% (59 patients) reported a history of tobacco smoking. Diagnostically, UA was identified in 64.6% (146 patients), STEMI in 18.6% (42 patients), and NSTEMI in 16.8% (38 patients). Among the patients with AMI, 4% (9 patients) were classified as Killip Class III and 3.1% (7 patients) as Killip Class IV, as presented in Table 1. In terms of the stent selection, the vast majority of patients (212/226, 93.8%) utilized self-funded new-generation drug-eluting stents (DESs), while approximately 15.5% used bare-metal stents (BMSs) covered by Taiwan’s National Health Insurance (NHI). Before inclusion in this study, 153 patients (67.7%) were receiving ACEI, ARBs, or sacubitril/valsartan (Entresto); 144 patients (63.7%) were on β-blockers; and 178 patients (78.8%) were taking statins.

The baseline characteristics of the treated lesions and procedural details are presented in Table 2. A total of 448 lesions were treated, of which 10 (2.2%) were located in the left main coronary artery, 199 (44.4%) in the left anterior descending coronary artery, 69 (15.4%) were located in the left circumflex coronary artery, and 170 (38.0%) in the right coronary artery. The lesions were classified according to the American College of Cardiology (ACC)/American Heart Association (AHA) lesions classification system [22], with 139 (31.0%) identified as type B lesions and 309 (69.0%) as type C lesions. CTO was present in 19 (4.2%) lesions, and 21 (4.7%) involved bifurcations. The average lesion length and reference vessel diameter were 26.6 ± 12.1 mm and 3.1 ± 0.5 mm, respectively. Intravascular ultrasound (IVUS)-guided coronary stenting was performed in 322 lesions. Specifically, IVUS was utilized in 72.4% (113/156) of lesions in patients with an eGFR < 60 mL/min/1.73 m^2^ and 71.6% (209/292) of lesions in patients with an eGFR ≥ 60 mL/min/1.73 m^2^. Additionally, new-generation DESs were used in the majority of lesions (351/448, 78.3%).

### 3.2. Primary Efficacy, Primary Safety, and Secondary Safety Endpoints

Table 3 presents the incidences of cardiovascular events during prasugrel treatment. The incidence of the primary efficacy endpoint of MACEs (comprising all-cause mortality, non-fatal MI, TLR, non-fatal ischemic stroke, and ST) was 7.1% (16/226). TLR was the most frequently observed MACE, occurring in 6.2% (14/226) of the patients. Further analysis of the TLR incidence across different interventional treatments revealed that patients receiving BMS had the highest incidence of TLR at 14.3% (5/35), followed by those treated with DCB at 8.3% (1/12). The lowest incidence was observed in patients treated with new-generation DES, at 3.8% (8/212). Furthermore, this study recorded one case of cardiac death (0.4%), two cases of non-cardiac death (0.9%), and one case of non-fatal ischemic stroke (0.4%). Regarding safety events, which were characterized by major bleeding incidents categorized as BARC types 3 and 5, two patients (0.8%) experienced such events, both of which were classified as BARC type 3a gastrointestinal bleeding. The two patients continued to take aspirin and reduced-dose prasugrel (3.75 mg) for one year and did not experience any further bleeding events after being prescribed proton pump inhibitors (PPIs). Additionally, they did not encounter MACEs during the study period. The incidence of NACEs at one year was 8.0% (18/226).

We utilized a multivariate Cox proportional hazards regression model to calculate the HRs and 95% CIs for the selected risk factors, including age, BMI, and eGFR, as presented in Table 4 and Table 5. Another combination model, using body weight as an example, is outlined in Supplementary Tables S3 and S4. These analyses were adjusted for two additional factors, including gender, hypertension, hyperlipidemia, diabetes mellitus, and tobacco smoking.

Table 4 and Table 5 reveal that neither advanced age nor overweight (BMI 25–29 kg/m^2^) nor obesity (BMI ≥ 30 kg/m^2^) increased the risk of adverse events (e.g., MACEs, TLR, and NACEs). Nevertheless, these factors were associated with MACEs, TLR, and NACEs, primarily through the lens of the eGFR. Specifically, individuals with impaired renal function (eGFR < 60 mL/min/1.73 m^2^) were 4.03 times more likely (95% CI = 1.37–11.90, *p* = 0.01) to encounter MACEs compared to those with optimal renal function (eGFR ≥ 60 mL/min/1.73 m^2^). Under the same conditions, they had a 3.23-fold higher likelihood of experiencing TLR (95% CI = 1.04–10.07, *p* = 0.04) and a 5.56-fold higher likelihood of experiencing NACEs (95% CI = 1.94–15.92, *p* = 0.001). Supplementary Tables S3 and S4 reveal similar results to Table 4 and Table 5. Moreover, individuals with reduced renal function (eGFR < 60 mL/min/1.73 m^2^) had a 3.90-fold increased probability (95% CI = 1.33–11.42, *p* = 0.01) of experiencing MACEs, a 3.06-fold higher chance (95% CI = 1.00–9.41, *p* = 0.05) of facing TLR, and a 5.38-fold higher risk (95% CI = 1.90–15.25, *p* = 0.002) of NACEs compared to those with superior renal function (eGFR ≥ 60 mL/min/1.73 m^2^).

**Figure 2:**
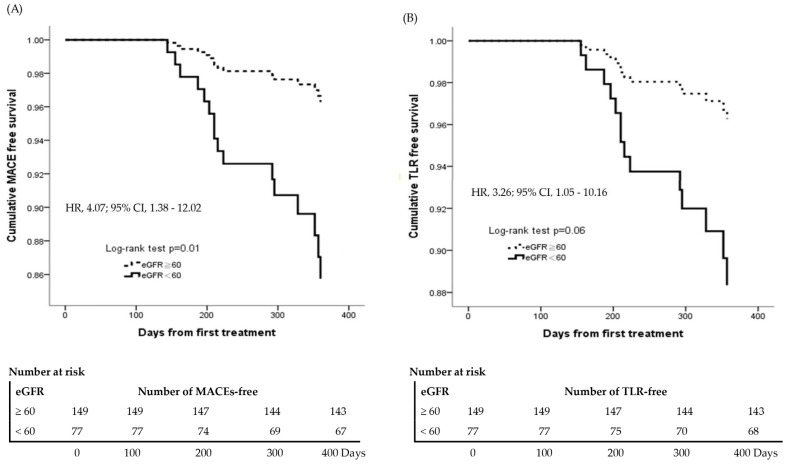
One-year cumulative event-free rates of study endpoints during prasugrel treatment. HRs were adjusted for age, BMI, gender, hypertension, hyperlipidemia, diabetes mellitus, and tobacco smoking. (**A**) MACE-free survival rates between eGFR < 60 and eGFR ≥ 60. (**B**) TLR-free survival rates between eGFR < 60 and eGFR ≥ 60. The definition of MACEs included cardiac death, MI, TLR, ischemic stroke, and stent thrombosis. TLR was defined as any need for repeated revascularization in the segment originally treated with new-generation DES, BMS, or DCB after documenting recurrent clinical ischemic symptoms and signs following the index procedure. HRs, hazard ratios; BMI, body mass index; MACEs, major adverse cardiac events; eGFR, estimated glomerular filtration rate; TLR, target lesion revascularization; MI, myocardial infarction; DES, drug-eluting stent; BMS, bare-metal stent; DCB, drug-coated balloon.

Based on these findings, individuals with an eGFR < 60 mL/min/1.73 m^2^ exhibited an elevated risk of MACEs and TLR compared to those with an eGFR ≥ 60 mL/min/1.73 m^2^. Therefore, the Kaplan–Meier method was employed to estimate the cumulative event-free rates for MACEs and TLR in both eGFR groups following one year of prasugrel treatment. These rates were then analyzed using the HR and 95% CI. Figure 2 illustrates that, after adjusting for age, BMI, gender, hypertension, hyperlipidemia, diabetes mellitus, and tobacco smoking, patients with an eGFR < 60 mL/min/1.73 m^2^ had a significantly lower cumulative incidence of MACE-free (Figure 2A: HR = 4.07, 95% CI = 1.38–12.02, *p* = 0.01) and TLR-free (Figure 2B: HR = 3.26, 95% CI = 1.05–10.16, *p* = 0.06) survival compared to those with an eGFR ≥ 60 mL/min/1.73 m^2^. Similar results are observed in Figure S2, where body weight was used instead of BMI. Patients with an eGFR < 60 mL/min/1.73 m^2^ had a lower cumulative incidence of MACE-free (Supplementary Figure S2A: HR = 3.91, 95% CI = 1.34–11.46, *p* = 0.01) and TLR-free (Supplementary Figure S2B: HR = 3.08, 95% CI = 1.00–9.48, *p* = 0.06) survival compared to those with an eGFR ≥ 60 mL/min/1.73 m^2^. Accounting for the competing risk of death is an important consideration in survival analysis for outcomes other than all-cause mortality. The incidence of MACEs compared with the competing risk (death before MACEs), is shown in Supplementary Figure S1.

## 4. Discussion

This single-center study evaluated the efficacy and safety of reduced-dose prasugrel (LD/MD: 20/3.75 mg) in Taiwanese ACS patients undergoing PCI. The key findings were as follows. (1) The one-year cumulative incidence of MACEs was 7.1%, predominantly due to TLR, which had a one-year incidence of 6.2%. In addition, the one-year cumulative incidence of NACEs was 8.0%. (2) Major bleeding events (BARC type 3 or 5) occurred in 0.8% of patients. (3) The hazard rates and one-year survival rates for MACEs, TLR, and NACEs were consistent across different ages, BMIs, and body weights. (4) Patients with a reduced eGFR (eGFR < 60 mL/min/1.73 m^2^) had a higher risk of MACEs, TLR, and NACEs compared to those with an eGFR ≥ 60 mL/min/1.73 m^2^.

Prasugrel and ticagrelor, as newer P2Y12 inhibitors, offer better cardiovascular outcomes compared to clopidogrel but carry a higher bleeding risk [7,23]. The TRITON-TIMI 38 trial highlighted that prasugrel (LD: 60 mg; daily MD: 10 mg daily) had no net benefit for patients aged 75 or older or those under 60 kg, and it was harmful to those with a history of ischemic stroke or TIA. Additionally, patients with these risk factors experienced higher bleeding rates [7]. East Asian populations often have higher average ages and lower body weights, so dose adjustments are important [24]. A Japanese Phase II study found that reduced-dose prasugrel (LD/MD: 20/3.75 mg) had lower bleeding risks and strong antiplatelet effects than clopidogrel (LD/MD: 300/75 mg) [8]. The PRASFIT-ACS study [13], which excluded those with ischemic stroke or TIA, found reduced-dose prasugrel to be associated with a 23% reduction in the MACE risk and similar serious bleeding rates compared to clopidogrel. Post-marketing studies in Japan (PRASFIT-Practice I and II) reported low incidences of MACEs and ST with reduced-dose prasugrel [25,26]. The ISAR-REACT 5 trial recommended a reduced MD of prasugrel (5 mg daily) for patients aged ≥ 75 years or weighting < 60 kg, showing reduced ischemic events compared to ticagrelor, with similar major bleeding rates [5]. Genetic factors, such as CYP2C19 polymorphisms, affect clopidogrel efficacy [27], which newer P2Y12 inhibitors like prasugrel and ticagrelor address [28]. While Asian patients may have a higher predisposition to CYP2C19 loss-of-function mutations, leading to increased platelet reactivity and a higher risk of bleeding compared to Western populations [29], studies in Korean and Japanese populations have shown that reduced-dose prasugrel offers stable platelet inhibition with a lower risk of bleeding compared to clopidogrel [30,31].

Reduced-dose prasugrel was introduced in Taiwan at the end of 2018 [28]. This study provides observational data on its efficacy and safety in Taiwanese ACS patients undergoing PCI. (1) Incidence of events: In our 12-month follow-up, the incidence of MACEs was 7.1%, with ischemic events excluding TLR standing at 0.9%. This was slightly lower than rates in the TRITON-TIMI 38 [7] (9.9% in the standard-dose prasugrel group) and PRASFIT-ACS [13] (9.4% in the reduced-dose prasugrel group) trials. Yota Koyabu et al. observed low incidences of all-cause mortality (1.2%) and MACEs (1.6%) in patients receiving reduced-dose prasugrel, though new PCI devices might also have influenced the outcomes [32]. The incidence of NACEs was 8% in our study. Fujii et al. also demonstrated that DAPT with reduced-dose prasugrel (3.75 mg) was associated with a lower risk of NACEs (clopidogrel vs. prasugrel = 20.6% vs. 12.6%, *p* < 0.01) in ACS patients treated with PCI [33]. (2) MACE components: We found reduced incidences of cardiac mortality (0.4%), non-fatal MI (0.4%), non-fatal ischemic stroke (0.4%), ST (0.4%), and TLR (6.2%), with TLR mostly due to BMS implantation. A multicenter registry outcome report demonstrated that compared to BMS, the use of DES in Taiwanese ACS patients resulted in a significantly lower one-year MACE rate (DES vs. BMS: 10.2% vs. 15.6%, *p* < 0.001) [34]. Similarly, the LEADERS FREE trial showed that new-generation DES was associated with a lower incidence of the primary efficacy endpoint (clinically driven TLR) compared to BMS (5.1% vs. 9.8%; *p* < 0.001) [35]. As a result, the DES has rapidly supplanted the BMS worldwide due to its superior clinical benefits. However, despite the implementation of universal healthcare in Taiwan, socioeconomic disparities persist in using high-cost medical services, with the cost of DESs potentially contributing to these inequalities. A study by Raymond N. Kuo et al. found that patients in the highest income quintile were more likely to receive DESs (odds ratio: 2.23, 95% confidence interval: 2.06–2.47, *p* < 0.001) compared to those in the lowest income quintile in Taiwan [36]. (3) Comparison with other studies: Concerns regarding bleeding have been particularly pronounced in Taiwan and other East Asian countries. Our study reported a major bleeding incidence of 0.8% (2/226), which is lower than the rates observed in the PRASFIT-ACS (1.9%) study [13] and PRASFIT-Practice I (1.6%) study [25]. Our findings are consistent with the standard-dose prasugrel bleeding rates (0.9%) observed in Korean patients [37]. The safety profile of reduced-dose prasugrel in high-bleeding-risk patients (e.g., elderly or low body weight) aligns with recent findings, suggesting it is a viable option even in these populations [7,13,24]. Furthermore, the 41.6% of HBR patients (94/226) in our study was comparable to that in the CREDO-KYOTO registry (43%) [38] and the PRASFIT-Practice II study (40.1%) [26], both of which involved Japanese patients. East Asian ACS patients tend to experience excessive inhibition of platelet function by potent P2Y12 inhibitors, significantly increasing the risk of major bleeding, suggesting a different ischemia/bleeding tradeoff to Caucasians [39]. Masanobu Ohya et al. observed that elderly (≥75 years), low body weight (≤50 kg), and renal insufficiency (eGFR ≤ 30 mL/min/1.73 m^2^) were independent predictors of in-hospital major bleeding in ACS patients [40]. However, the two patients who experienced major bleeding had a gastrointestinal hemorrhage in our study. Both patients belonged to the HBR group. The primary major bleeding endpoint was 2.1% (2/94) in the HBR group and no incident (0/132) in the non-HBR group. The two patients continued taking aspirin and reduced-dose prasugrel (3.75 mg) for one year with no further bleeding events after initiating PPIs. Additionally, the two patients did not encounter MACEs during the study period. The TRANSLACE-ACS study also demonstrated that, compared to clopidogrel, PPIs did not significantly increase the risk of MACEs or bleeding associated with prasugrel use in AMI patients treated with PCI [41]. Therefore, our study confirmed the safety of reduced-dose prasugrel (3.75 mg) in the treatment of Taiwanese ACS patients undergoing PCI regardless of HBR or non-HBR status. (4) Impact of patient factors: Factors such as age, body weight, BMI, and renal function affected clinical outcomes [42,43,44]. A systemic review and meta-analysis found that potent P2Y12 inhibitors like prasugrel or ticagrelor had a similar MACE risk compared to clopidogrel in elderly ACS patients but increased the major bleeding risk [45]. The TRITON-TIMI 38 trial showed no clinical benefit of standard-dose prasugrel in patients aged ≥ 75 years or with lower body weight [28]. In this study, no significant differences were observed in the MACE, TLR, NACE, and cumulative MACE-free and TLR-free survival rates among Taiwanese ACS patients undergoing PCI regardless of age (<75 or ≥75 years), BMI (<25 or ≥25 kg/m^2^), and body weight (<60 or ≥60 kg) after 12 months of reduced-dose prasugrel therapy. Overall, reduced-dose prasugrel demonstrates effectiveness and safety for Taiwanese ACS patients undergoing PCI, even considering high-bleeding-risk factors.

Our study supports the efficacy of reduced-dose prasugrel across different demographics, showing that ACS patients with a reduced eGFR (<60 mL/min/1.73 m^2^) had higher MACE, TLR, and NACE risks and lower cumulative survival rates compared to those with a higher eGFR (≥60 mL/min/1.73 m^2^) after 12 months of therapy. CKD is a significant risk factor for various cardiovascular diseases. A retrospective observational study found that a reduced eGFR was associated with an increased risk of MACEs [12]. Besides the risk of MACEs, the HORIZONS-AMI trial also demonstrated that STEMI patients with CKD had significantly higher rates of NACEs than those without CKD [46]. Morbidity in the kidneys could lead to cardiovascular morbidity, including a close interrelation between these two vital organs. Additional risk factors, such as anemia, volume overload, and systemic inflammation, also played critical roles in the risk of MACEs among CKD patients. Data from a multicenter registry that investigated the one-year clinical outcomes in CAD patients undergoing PCI with new-generation DES revealed that patients with CKD (eGFR < 60 mL/min/1.73 m^2^) and ESRD had a higher risk of MACEs, with ORs of 2.34 and 1.59, compared to those with normal renal function [47]. Therefore, Jun Pil Yun et al. demonstrated that patients with impaired renal function undergoing PCI for ACS were at a high risk of both ischemic and bleeding events and prasugrel dose de-escalation proved beneficial regardless of the baseline renal function in these patients [48]. Additionally, patients with CKD exhibited more complex, calcified, and extensive CAD compared to those without CKD. Therefore, IVUS-guided PCI may offer a more precise assessment of the lesion morphology and enhance the stent expansion and optimization, potentially leading to improved cardiovascular outcomes compared to angiography-guided PCI in the era of new-generation DES. In our study, IVUS was indeed frequently used for assistance (Table 2). Furthermore, IVUS-guided PCI consistently resulted in favorable outcomes regardless of the eGFR level and ESRD [49]. Among the 16 patients with major adverse cardiovascular events (MACEs) in our study, four had STEMI (9.5% of STEMI patients), three had NSTEMI (7.9% of NSTEMI patients), and nine had UA (6.2% of UA patients). The STEMI patients had the highest proportion of MACEs. Additionally, a sustained systemic and local inflammatory response, accompanied by edema, has been histologically observed in non-infarcted myocardium (NIM) in animal models of STEMI. Luca Bergamaschi et al. used cardiac magnetic resonance (CMR) to investigate the relationship between NIM and the surrounding tissues (including the liver, spleen, and pectoralis muscle) as well as MACEs in STEMI patients without prior cardiovascular events. Their findings indicated that elevated NIM T2 values (>45 ms) were associated with an increased risk of MACEs compared to patients with NIM T2 values ≤ 45 ms [50]. These results highlight the potential of NIM T2 values as a valuable tool for predicting cardiovascular outcomes in ACS patients undergoing PCI.

### Study Limitations

First, this study was a retrospective, single-center, observational analysis with a relatively small sample size of ACS patients, limiting our findings’ generalizability. Second, the non-randomized design of this study lacked a concurrent control group; however, we mitigated this limitation by comparing our findings with international evidence-based research. Third, the Veterans Affairs Extended DAPT study demonstrated that patients who underwent PCI with new-generation DES exhibited lower long-term risks of all-cause mortality, cardiac death, MI, and bleeding events when DAPT was discontinued after nine months [51]. Our study focused on the outcomes of Taiwanese ACS patients who received DAPT for one year after PCI; therefore, we excluded patients who received DAPT for only 6 months (49 patients) or 9 months (79 patients) following PCI, which may have introduced selection bias and influenced the results. However, 12-month DAPT remains the only Class I recommendation for patients with ACS undergoing PCI. Notably, the average DAPT duration in the CURE trial was 9 months, rather than the recommended 12 months [10]. Several studies have also provided evidence supporting the rationale for DAPT de-escalation or shorter DAPT durations in ACS patients undergoing PCI [48,52]. Given these findings, a future analysis comparing the clinical outcomes of different DAPT durations in ACS patients undergoing PCI would be valuable. Fourth, although Taiwan’s NHI benefits plan has substantially improved access to DESs, socioeconomic disparities remain widespread. Yong et al. observed that lower-income individuals are less likely to receive DESs among patients with ACS [53]. This disparity may stem from patients with higher incomes being more likely to have insurance that covers the costs of expensive procedures. Fifth, the limited number of events may have reduced the statistical power to detect clinical hard endpoints. Consequently, further studies are needed to address these limitations. Despite these constraints, our study demonstrates that reduced-dose prasugrel is safe and effective for Taiwanese patients with ACS undergoing PCI. Fifth, the current regulations set by Taiwan’s National Health Insurance Administration, Ministry of Health and Welfare, and the Taiwan Society of Cardiology (TSOC) guidelines for managing ACS, permit only the use of a reduced dose of prasugrel. These regulatory constraints prevented us from directly comparing the efficacy and safety of full-dose (LD/MD: 60 mg/10 mg) and reduced-dose prasugrel. Nevertheless, we conducted a meta-analysis comparing the efficacy and safety of 10 mg/3.75 mg prasugrel with clopidogrel in ACS patients (see Supplementary Figure S3 and Table S5). The results show that reduced-dose prasugrel is as effective as full-dose prasugrel, with a better safety profile for ACS patients.

## 5. Conclusions

The one-year follow-up results confirmed that the reduced-dose prasugrel regimen (LD/MD: 20 mg/3.75 mg) is both safe and effective for Taiwanese ACS patients undergoing PCI. Notably, patients with an eGFR < 60 mL/min/1.73 m^2^ exhibited a higher risk of MACEs, LTR, and NACEs, as well as lower cumulative MACE-free and TLR-free survival rates, compared to those with an eGFR ≥ 60 mL/min/1.73 m^2^.

## Figures and Tables

**Table 1:** Baseline clinical characteristics of patients.

Characteristic	Total, n (%) (N = 226)
Sex	
Male	182 (80.5)
Age (years)	
≥75	51 (22.6)
Mean ± SD	66.1 ± 11.1
BMI (kg/m^2^)	
≥25	129 (57.1)
Mean ± SD	26.5 ± 3.8
Weight (kg)	
≥60	170 (75.2)
Mean ± SD	70 ± 11.7
Medical history	
Prior MI	25 (11.1)
Prior ischemic stroke	24 (10.6)
Prior PCI	9 (4.0)
Prior CABG	4 (1.8)
Arial fibrillation	8 (3.5)
ESRD	17 (7.1)
ARC-HBR	94 (41.6)
Risk factors	
Hypertension	117 (51.8)
Hyperlipidemia	164 (72.6)
Diabetes mellitus	88 (38.9)
Tobacco smoking	59 (26.1)
Complex PCI	
3 vessels treated	126 (55.8)
≥3 stents implanted	60 (26.5)
Bifurcation with 2 stents implanted	5 (2.2%)
Total stent length > 60 mm	39 (17.3%)
Left main PCI	10 (4.4%)
Chronic total occlusion	17 (7.5%)
Diagnosis	
ST-segment elevation MI	42 (18.6)
Non-ST-segment elevation MI	38 (16.8)
Unstable angina pectoris	146 (64.6)
Killip classification	
Class I	56 (24.8)
Class II	8 (3.5)
Class III	9 (4.0)
Class IV	7 (3.1)
Stent type	
New-generation DES	212 (93.8)
BMS	35 (15.5%)
DCB	12 (5.3%)
eGFR (mL/min/1.73 m^2^) < 60	77 (34.1)
LVEF (%)	58 ± 15.4
Medication	
ACEI, ARBs, or sacubitril/valsartan (Entresto)	153 (67.7)
β-blockers	144 (63.7)
Statins	178 (78.8)

Data are n (%) or mean ± SD. SD, standard deviation; BMI, body mass index; MI, myocardial infarction; PCI, percutaneous coronary intervention; CABG, coronary artery bypass grafting; ESRD, end-stage renal disease; ARC-HBR, Academic Research Consortium for High Bleeding Risk; DES, drug-eluting stent; BMS, bare-metal stent; DCB, drug-coated balloon; eGFR, estimated glomerular filtration rate; LVEF, left ventricular ejection fraction; ACEI, angiotensin-converting enzyme inhibitor; ARB, angiotensin receptor blocker.

**Table 2:** Baseline characteristics of treated lesions and procedures.

Characteristic	Total, n (%) (Treated Lesions = 448)
Treated lesions	
Left main coronary artery	10 (2.2)
Left anterior descending coronary artery	199 (44.4)
Left circumflex coronary artery	69 (15.4)
Right coronary artery	170 (38.0)
ACC/AHA lesion type	
A	0 (0.0)
B	139 (31.0)
C	309 (69.0)
Chronic total occlusion	19 (4.2)
Bifurcation	21 (4.7)
Length of lesion (mm)	26.6 ± 12.1
RVD (mm)	3.1 ± 0.5
IVUS	322 (71.9)
Lesions in patient’s eGFR < 60 mL/min/1.73 m^2^	156 (34.8)
IVUS (+)	113
IVUS (-)	43
Lesions in patient’s eGFR ≥ 60 mL/min/1.73 m^2^	292 (65.2)
IVUS (+)	209
IVUS (-)	83
Length of stent (mm)	30.3 ± 11
MLD (mm)	
Pre-PCI	0.5 ± 0.4
Post-PCI	2.7 ± 0.5
Diameter stenosis (%)	
Pre-PCI	85.1 ± 11.2
Post-PCI	11.5 ± 9.3
Stent type	
New-generation DES	351 (78.3)
BMS	68 (15.2)
DCB	29 (6.5)

Data are n (%) or mean ± SD. ACC, American College of Cardiology; AHA, American Heart Association; SD, standard deviation; RVD, reference vessel diameter; IVUS, intravascular ultrasound; eGFR, estimated glomerular filtration rate; MLD, minimal lumen diameter; PCI, percutaneous coronary intervention; DES, drug-eluting stent; BMS, bare-metal stent; DCB, drug-coated balloon.

**Table 3:** Clinical outcomes during 12-month follow-up.

	12 Months, Total, n (%) (N = 226)
MACE	16 (7.1)
All-cause mortality	3 (1.3)
Cardiac	1 (0.4)
Non-cardiac	2 (0.9)
Non-fatal MI	1 (0.4)
Culprit (target vessel MI)	1 (0.4)
Non-culprit	0 (0.0)
TLR	14 (6.2)
Proportion of patients receiving new-generation DES	8/212 (3.8%)
Proportion of patients receiving BMS	5/35 (14.3%)
Proportion of patients receiving DCB	1/12 (8.3%)
Non-fatal ischemic stroke	1 (0.4)
Stent thrombosis	1 (0.4)
Major bleeding: BARC types 3 and 5	
Type 3a	2 (0.8)
NACE	18 (8.0)

Data on n (%). MACE, major adverse cardiac events; MI, myocardial infarction; TLR, target lesion revascularization; DES, drug-eluting stent; BMS, bare-metal stent; DCB, drug-coated balloon; BARC, Bleeding Academic Research Consortium; NACE, net adverse clinical events.

**Table 4:** The individual effect of age, BMI, and eGFR on the risk of MACEs and NACEs.

	Unadjusted Model		Adjusted Model *	
Variables	Hazard Ratio (95% CI)	*p*-Value	Hazard Ratio (95% CI)	*p*-Value
	**MACEs**			
Age (years)				
Age < 75	Reference		Reference	
Age ≥ 75	0.54 (0.12–2.40)	0.42	0.34 (0.07–1.58)	0.17
BMI (kg/m^2^)				
BMI ≥ 25	Reference		Reference	
BMI < 25	0.67 (0.25–1.79)	0.42	0.66 (0.24–1.80)	0.42
eGFR (mL/min/1.73 m^2^)				
eGFR ≥ 60	Reference		Reference	
eGFR < 60	3.28 (1.19–9.03)	0.02	4.03 (1.37–11.90)	0.01
	**NACEs**			
Age (years)				
Age < 75	Reference		Reference	
Age ≥ 75	0.486 (0.11–2.12)	0.336	0.25 (0.05–1.16)	0.077
BMI (kg/m^2^)				
BMI ≥ 25	Reference		Reference	
BMI < 25	0.808 (0.32–2.06)	0.654	0.74 (0.28–1.94)	0.54
eGFR (mL/min/1.73 m^2^)				
eGFR ≥ 60	Reference		Reference	
eGFR < 60	3.87 (1.45–10.32)	0.007	5.56 (1.94–15.92)	0.001

* Adjusted for another two risk factors, which also included gender, hypertension, hyperlipidemia, diabetes mellitus, and tobacco smoking. BMI, body mass index; eGFR, estimated glomerular filtration rate; MACE, major adverse cardiovascular events; NACE, net adverse clinical events.

**Table 5:** The individual effect of age, BMI, and eGFR on the risk of TLR.

	Unadjusted Model		Adjusted Model *	
Variables	Hazard Ratio (95% CI)	*p*-Value	Hazard Ratio (95% CI)	*p*-Value
Age (years)				
Age < 75	Reference		Reference	
Age ≥ 75	0.63 (0.14–2.82)	0.55	0.46 (0.1–2.2)	0.32
BMI (kg/m^2^)				
BMI ≥ 25	Reference		Reference	
BMI < 25	0.51 (0.18–1.47)	0.21	0.48 (0.16–1.42)	0.18
eGFR (mL/min/1.73 m^2^)				
eGFR ≥ 60	Reference		Reference	
eGFR < 60	2.65 (0.93–7.65)	0.07	3.23 (1.04–10.07)	0.04

* Adjusted for another two risk factors, which also included gender, hypertension, hyperlipidemia, diabetes mellitus, and tobacco smoking. BMI, body mass index; eGFR, estimated glomerular filtration rate; TLR, target lesion revascularization.

## Data Availability

Data are unavailable due to privacy or ethical restrictions.

## Supplementary Materials

The following supporting information can be downloaded at https://www.mdpi.com/article/10.3390/jcm13237221/s1, Figure S1: Incidence of MACEs compared with the competing risk (death before MACEs); Figure S2: One-year cumulative event-free rates of study endpoints during prasugrel treatment; Figure S3: Comparing the efficacy and safety of prasugrel and clopidogrel through a meta-analysis. Table S1: Predictors of MACEs at 12 months; Table S2: Predictors of TLR at 12 months; Table S3: The individual effect of age, body weight, and eGFR on the risk of MACEs and NACEs; Table S4: The individual effect of age, body weight, and eGFR on the risk of TLR. Table S5: Details for extracting data from randomized controlled trials [7,8,13,31,40,54,55,56,57,58,59,60,61,62,63,64].
