# Supplementary material for: Clinical Efficacy and Safety of Reduced-Dose Prasugrel After Percutaneous Coronary Intervention for Taiwanese Patients with Acute Coronary Syndromes
Source: J Clin Med. 2024 Nov 27;13(23):7221. doi: 10.3390/jcm13237221 (PMC11642127; doi:10.3390/jcm13237221)
Supplement: Supplementary file 1 [file jcm-13-07221-s001.zip › jcm-3243694-supplementary.pdf]

(A)

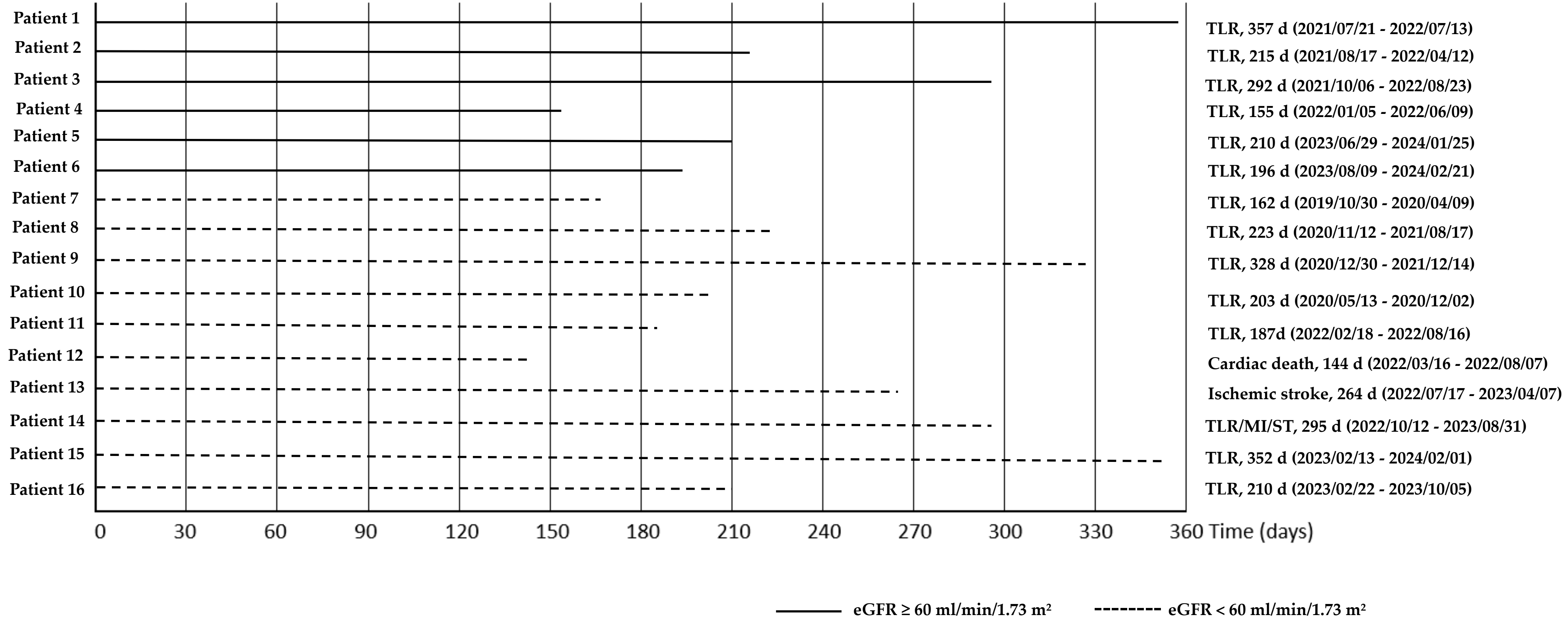

(B)

	Time (days)	0	100	200	300	400
Overall	MACE (event of interest)	0	0	5	8	3
	Non-cardiac death (competing event)	0	2	0	0	0
	SDH	0/226	2/226	5/226	8/221	3/213
	CSH	0/226	2/226	5/224	8/219	3/211
eGFR ≥ 60	MACE (event of interest)	0	0	2	3	1

	Non-cardiac death (competing event)	0	0	0	0	0
	SDH	0/149	0/149	2/149	3/147	1/144
	CSH	0/149	0/149	2/149	3/147	1/144
eGFR < 60	MACE (event of interest)	0	0	3	5	2
	Non-cardiac death (competing event)	0	2	0	0	0
	SDH	0/77	0/77	3/77	5/74	2/69
	CSH	0/77	2/77	3/75	0/72	0/72

(C)

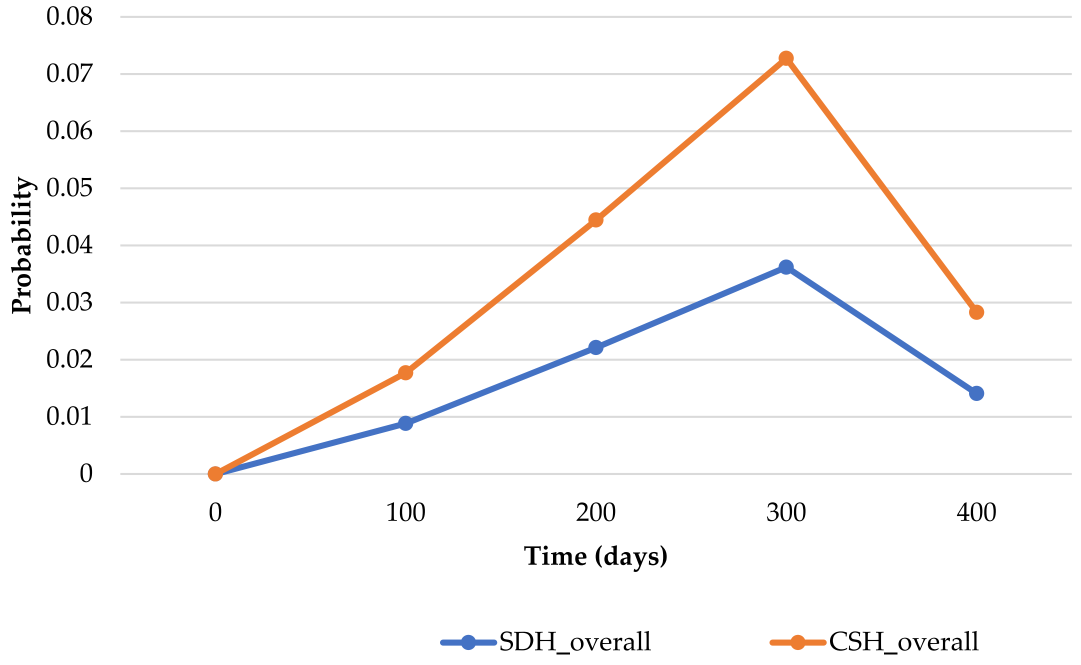

(D)

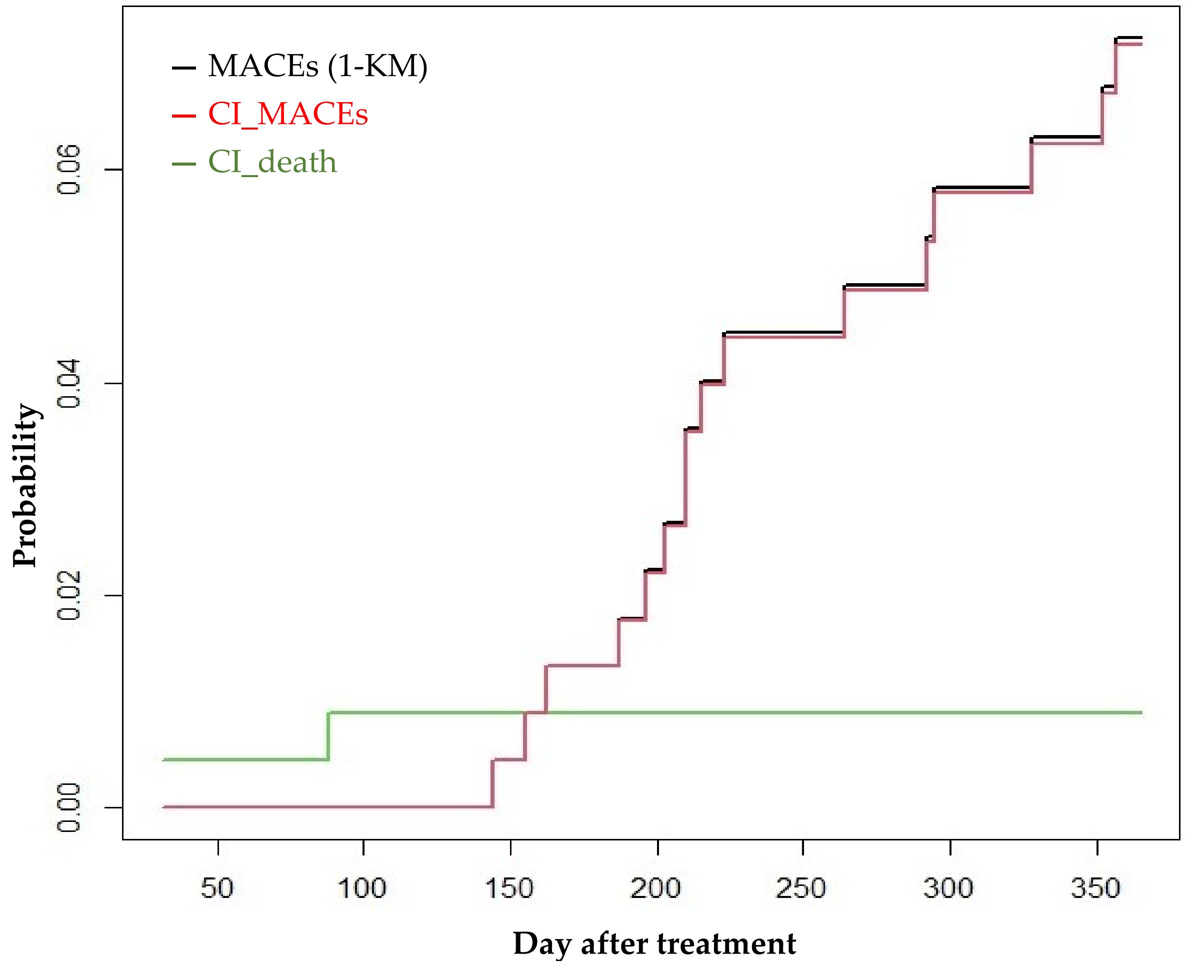

Supplementary Figure S1. Incidence of MACEs compared with the competing risk (death before MACEs). (A) Sixteen Taiwanese ACS patients experienced MACE events during the observation period. The definition of MACE includes cardiac death, myocardial infarction (MI), target lesion revascularization (TLR), ischemic stroke, and stent thrombosis (ST). (B) Document the MACEs and competitive events among 226 ACS patients. (C) The cause-specific and sub-distribution hazard models estimate probabilities on a 100-day basis. CSH refers to the cause-specific hazard function, while SDH refers to the sub-distribution hazard function. (D) Cumulative incidence rates of MACEs and mortality risk.

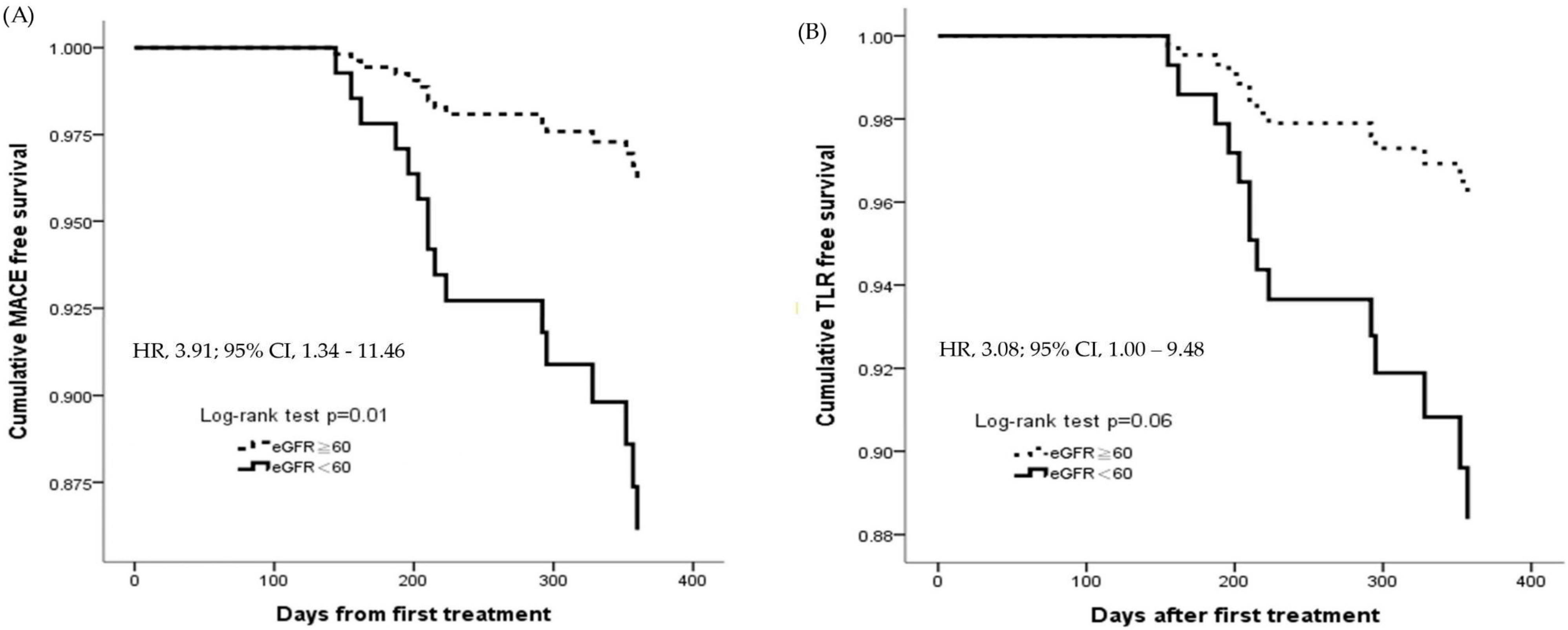

Number at risk

eGFR	Number of MACEs-free				
≥ 60	149	149	147	144	143
< 60	77	77	74	69	67
	0	100	200	300	400 Days

Number at risk

eGFR	Number of TLR-free				
≥ 60	149	149	147	144	143
< 60	77	77	75	70	68
	0	100	200	300	400 Days

Supplementary Figure S2. One-year cumulative event-free rates of study endpoints during prasugrel treatment. HRs were adjusted for age, body weight, gender, hypertension, hyperlipidemia, diabetes mellitus, and tobacco smoking. (A) MACE-free survival rates between eGFR < 60 and eGFR ≥ 60 ml/min/1.73m^2. (B) TLR-free survival rates between eGFR < 60 and eGFR ≥ 60 ml/min/1.73m^2. The definition of MACEs included cardiac death, MI, TLR, ischemic stroke, and stent thrombosis. TLR was defined as any need for repeated revascularization in the segment originally treated with new-generation DES, BMS, or DCB after documenting recurrent clinical ischemic symptoms and signs following the index procedure. HRs, hazard ratios; MACEs, major adverse cardiac events; eGFR, estimated glomerular filtration rate; TLR, target lesion revascularization; MI, myocardial infarction; DES, drug-eluting stent; BMS, bare-metal stent; DCB, drug-coated balloon.

(A)

Study or Subgroup	Prasugrel Events	Prasugrel Total	Clopidogrel Events	Clopidogrel Total	Weight	Odds Ratio M-H, Random, 95% CI
Murphy 2008	58	529	115	674	19.4%	0.60 [0.43, 0.84]
Park 2018	15	618	18	617	11.0%	0.83 [0.41, 1.66]
Pufulete 2022	57	406	337	1023	20.2%	0.33 [0.24, 0.45]
Roe 2012	621	4663	648	4663	24.6%	0.95 [0.85, 1.07]
Wiviott 2007	643	6813	781	6795	24.8%	0.80 [0.72, 0.90]
**Total (95% CI)**		**13029**		**13772**	**100.0%**	**0.66 [0.49, 0.90]**
Total events	1394		1899			

Heterogeneity: Tau² = 0.09; Chi² = 42.23, df = 4 (P < 0.00001); I² = 91%
Test for overall effect: Z = 2.64 (P = 0.008)

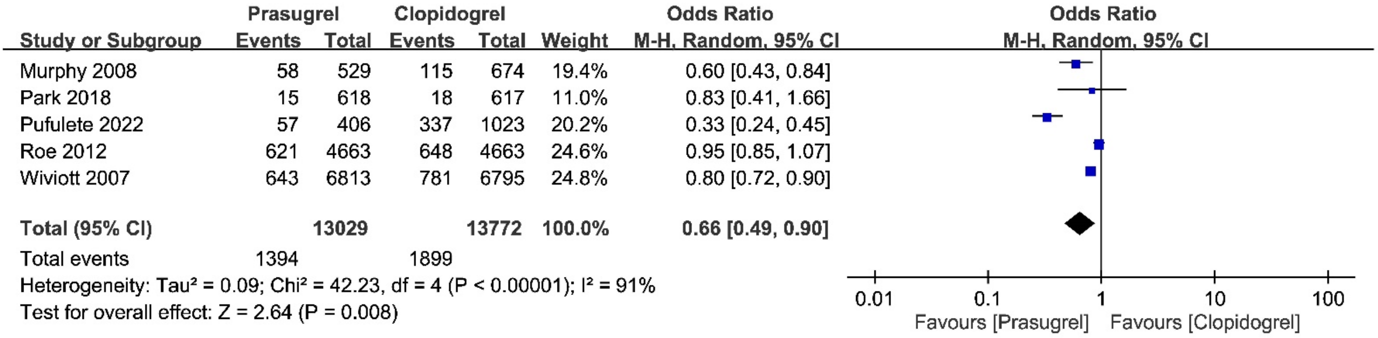

(B)

Study or Subgroup	Experimental Events	Experimental Total	Control Events	Control Total	Weight	Odds Ratio M-H, Fixed, 95% CI
Isshiki 2014	15	370	25	372	8.3%	0.59 [0.30, 1.13]
Kimura 2015	6	141	8	140	2.7%	0.73 [0.25, 2.17]
Kitagawa 2020	9	216	8	223	2.6%	1.17 [0.44, 3.09]
Kitano 2020	5	37	4	38	1.2%	1.33 [0.33, 5.39]
Kitazono 2023	45	1337	58	1352	19.4%	0.78 [0.52, 1.16]
Kitazono 2023-2	8	118	8	112	2.7%	0.95 [0.34, 2.61]
Ohya 2018	2	487	6	192	3.0%	0.13 [0.03, 0.64]
Saito 2014	74	685	84	678	26.2%	0.86 [0.61, 1.19]
Shibata 2021	17	204	35	330	8.5%	0.77 [0.42, 1.41]
Yasuda 2019	177	2607	46	462	25.4%	0.66 [0.47, 0.93]
**Total (95% CI)**		**6202**		**3899**	**100.0%**	**0.75 [0.63, 0.90]**
Total events	358		282			

Heterogeneity: Chi² = 8.03, df = 9 (P = 0.53); I² = 0%
Test for overall effect: Z = 3.20 (P = 0.001)

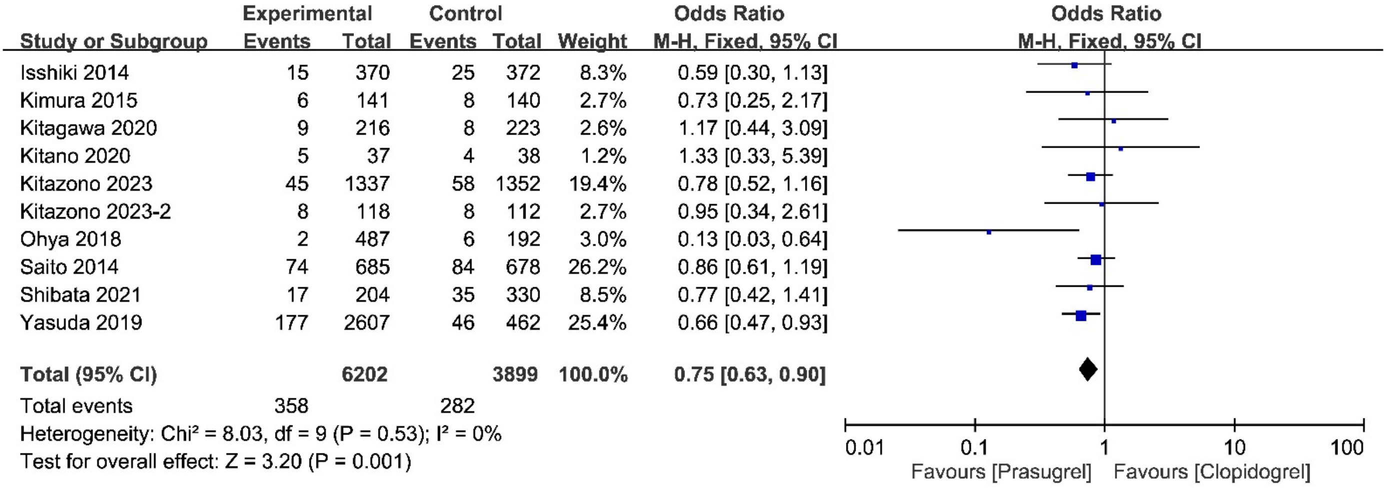

(C)

Study or Subgroup	Prasugrel Events	Prasugrel Total	Clopidogrel Events	Clopidogrel Total	Weight	Odds Ratio M-H, Random, 95% CI
Murphy 2008	13	529	12	674	14.7%	1.39 [0.63, 3.07]
Park 2018	34	637	17	637	18.8%	2.06 [1.14, 3.72]
Pufulete 2022	9	406	63	1023	16.3%	0.35 [0.17, 0.70]
Roe 2012	58	4623	48	4617	23.7%	1.21 [0.82, 1.78]
Wiviott 2007	146	6813	111	6795	26.5%	1.32 [1.03, 1.69]
**Total (95% CI)**		**13008**		**13746**	**100.0%**	**1.14 [0.74, 1.75]**
Total events	260		251			

Heterogeneity: Tau² = 0.17; Chi² = 15.75, df = 4 (P = 0.003); I² = 75%
Test for overall effect: Z = 0.58 (P = 0.56)

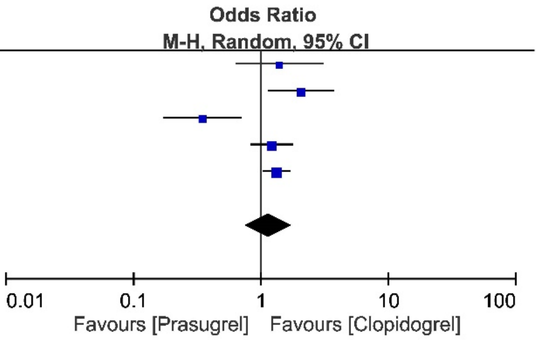

(D)

Study or Subgroup	Prasugrel Events	Prasugrel Total	Clopidogrel Events	Clopidogrel Total	Weight	Odds Ratio M-H, Fixed, 95% CI
Isshiki 2014	0	370	8	372	7.0%	0.06 [0.00, 1.01]
Kimura 2015	0	141	2	140	2.1%	0.20 [0.01, 4.11]
Kitagawa 2020	0	216	0	223		Not estimable
Kitazono 2023	2	1337	1	1351	0.8%	2.02 [0.18, 22.33]
Kitazono 2023-2	1	118	0	112	0.4%	2.87 [0.12, 71.25]
Kuno 2024	9	659	109	4733	21.6%	0.59 [0.30, 1.16]
Ohya 2018	3	487	5	192	5.9%	0.23 [0.05, 0.98]
Saito 2014	13	685	15	678	12.1%	0.86 [0.40, 1.81]
Shibata 2021	4	204	3	330	1.8%	2.18 [0.48, 9.84]
Yasuda 2019	99	2607	36	462	48.3%	0.47 [0.31, 0.69]
**Total (95% CI)**		**6824**		**8593**	**100.0%**	**0.55 [0.41, 0.73]**
Total events	131		179			

Heterogeneity: Chi² = 11.59, df = 8 (P = 0.17); I² = 31%
Test for overall effect: Z = 4.05 (P < 0.0001)

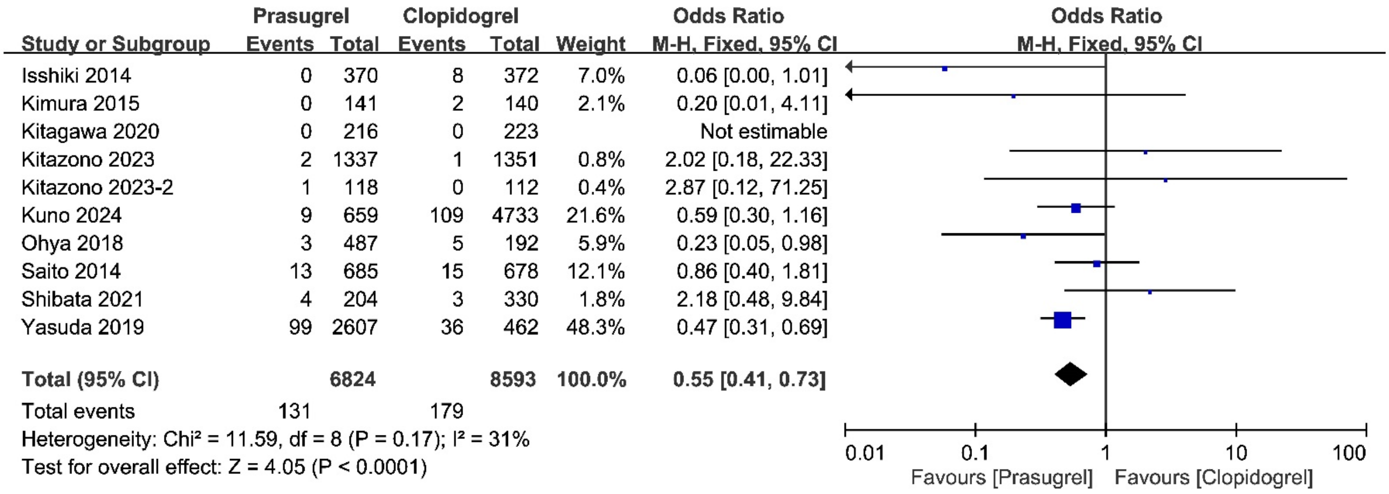

Supplementary Figure S3. Comparing the efficacy and safety of prasugrel and clopidogrel through a meta-analysis. (A) MACEs, 10-mg Prasugrel vs. Clopidogrel; (B) MACEs, 3.75-mg Prasugrel vs. Clopidogrel; (C) Major bleeding, 10-mg Prasugrel vs. Clopidogrel; and (D) Major bleeding, 3.75-mg Prasugrel vs. Clopidogrel.

Supplementary Table S1. Predictors of MACEs at 12 months

	Univariate analysis		
Variables	HR	95% CI	*p*-value
Age ≥ 75 years	1.84	(0.42 – 8.09)	0.421
Male	3.33	(0.44 – 25.2)	0.244
Weight < 60 Kg	1.31	(0.37 – 4.61)	0.671
BMI < 25 Kg/m^2	1.50	(0.56 – 4.0)	0.422
eGFR < 60 ml/min/1.73m^2	3.28	(1.19 – 9.03)	0.022
ARC - HBR	2.35	(0.85 – 6.46)	0.099
Hypertension	1.21	(0.45 – 3.26)	0.700
Hyperlipidemia	1.09	(0.35 – 3.39)	0.880
DM	1.97	(0.73 – 5.28)	0.180
Tobacco smoking	1.68	(0.48 – 5.90)	0.419
In-hospital medication			
Statin	1.81	(0.41 – 7.97)	0.432
ACEI/ARB/Entresto	1.80	(0.67 – 4.84)	0.243
β - blockers	2.53	(0.72 – 8.90)	0.147
Complex PCI	34.70	(0.36 – 3315.98)	0.127
IVUS	1.20	(0.39 – 3.74)	0.750

Supplementary Table S2. Predictors of TLR at 12 months

Variables	Univariate analysis		
	HR	95% CI	*p*-value
Age ≥ 75 years	1.59	(0.35 – 7.09)	0.547
Male	2.92	(0.38 – 22.3)	0.302
Weight < 60 Kg	1.12	(0.31 – 4.02)	0.863
BMI < 25 Kg/m^2	1.97	(0.68 – 5.71)	0.210
eGFR < 60 ml/min/1.73m^2	2.65	(0.92 – 7.65)	0.071
ARC - HBR	1.90	(0.66 – 5.47)	0.236
Hypertension	1.69	(0.57 – 5.04)	0.348
Hyperlipidemia	1.34	(0.37 – 4.81)	0.653
DM	1.53	(0.23 – 1.86)	0.423
Tobacco smoking	1.41	(0.39 – 5.05)	0.599
In-hospital medication			
Statin	1.55	(0.35 – 6.91)	0.569
ACEI/ARB/Entresto	1.86	(0.64 – 5.36)	0.252
β - blockers	2.13	(0.59 – 0.64)	0.246
Complex PCI	34.85	(0.27 – 4506.25)	0.152
IVUS	1.49	(0.42 – 5.36)	0.539

Supplementary Table S3. The individual effect of age, body weight, and eGFR on the risk of MACEs and NACEs

	Unadjusted model		Adjusted model *	
Variables	Hazard ratio (95% CI)	*p*-value	Hazard ratio (95% CI)	*p*-value
	MACEs			
**Age (years)**				
Age < 75	Reference		Reference	
Age ≥ 75	0.54 (0.12 – 2.40)	0.42	0.33 (0.07 – 1.54)	0.16
**Body weight (kg)**				
Body weight ≥ 60	Reference		Reference	
Body weight < 60	1.31 (0.37 – 4.61)	0.67	1.22 (0.33 – 4.49)	0.77
**eGFR (ml/min/1.73m^2)**				
eGFR ≥ 60	Reference		Reference	
eGFR < 60	3.28 (1.19 – 9.03)	0.02	3.90 (1.33 – 11.42)	0.01
	NACEs			
**Age (years)**				
Age < 75	Reference		Reference	
Age ≥ 75	0.49(0.11-2.12)	0.336	0.25(0.05-1.16)	0.08
**Body weight (kg)**				
Body weight ≥ 60	Reference		Reference	
Body weight < 60	1.48(0.43-5.13)	0.535	1.30(0.36-4.69)	0.69
**eGFR (ml/min/1.73m^2)**				
eGFR ≥ 60	Reference		Reference	
eGFR < 60	3.87(1.45-10.32)	0.007	5.38(1.90-15.25)	0.002

*Adjusted for another two risk factors, which also included gender, hypertension, hyperlipidemia, diabetes mellitus, and tobacco smoking. eGFR, estimated glomerular filtration rate; MACEs, major adverse cardiovascular events; NACEs, net adverse clinical events.

Supplementary Table S4. The individual effect of age, body weight, and eGFR on the risk of TLR

	Unadjusted model		Adjusted model *	
Variables	Hazard ratio (95% CI)	*p*-value	Hazard ratio (95% CI)	*p*-value
**Age (years)**				
Age < 75	Reference		Reference	
Age ≥ 75	0.63 (0.14 – 2.82)	0.55	0.45 (0.09 – 2.14)	0.32
**Body weight (kg)**				
Body weight ≥ 60	Reference		Reference	
Body weight < 60	1.12 (0.31 – 4.02)	0.86	0.94 (0.25 – 3.52)	0.93
**eGFR (ml/min/1.73m^2)**				
eGFR ≥ 60	Reference		Reference	
eGFR < 60	2.65 (0.92 – 7.65)	0.07	3.06 (1.00 – 9.41)	0.05

*Adjusted for another two risk factors, which also included gender, hypertension, hyperlipidemia, diabetes mellitus, and tobacco smoking. eGFR, estimated glomerular filtration rate; TLR, target lesion revascularization.

Supplementary Table S5. Details for extracting data from randomized controlled trials

Dosage of Prasugrel	First author & Year & #ref	Sample size	Randomized groups	Clinical outcomes	Follow-up period
**1. Full-dose (10 mg)**					
	Wiviott, SD. (2007) # 7	13,608	**Prasugrel** (a 60-mg loading dose and a 10-mg daily maintenance dose) versus **Clopidogrel** (a 300-mg loading dose and a 75-mg daily maintenance dose).	**MACE**: Prasugrel (643/6813), Clopidogrel (781/6795). **Major bleeding**: Prasugrel (146/6813), Clopidogrel (111/6795).	6 - 15 months
	Murphy, SA. (2008) # 54	1,203	**Prasugrel** (60-mg LD/10-mg daily) versus **Clopidogrel** (300-mg LD/75-mg daily).	**MACE**: Prasugrel (58/529), Clopidogrel (115/674). **Major bleeding**: Prasugrel (13/529), Clopidogrel (12/674).	6 - 15 months
	Roe, MT. (2012) # 55	9,326	**Prasugrel** (60-mg LD/10-mg daily) versus **Clopidogrel** (300-mg LD/75-mg daily).	**MACE**: Prasugrel (621/4663), Clopidogrel (648/4663). **Major bleeding**: Prasugrel (58/4623), Clopidogrel (48/4617).	6 - 30 months
	Park, KH. (2018) # 56	4,421	**Prasugrel** (60-mg LD/10- or 5-mg daily) versus **Clopidogrel** (300-mg LD/75-mg daily).	**MACE**: Prasugrel (15/618), Clopidogrel (18/617). **Major bleeding**: Prasugrel (34/637), Clopidogrel (17/637).	6 months
	Pufulete, M. (2022) # 57	2,587	**Prasugrel** (60-mg LD/10-mg daily) versus **Clopidogrel** (300-mg LD/75-mg daily).	**MACE**: Prasugrel (57/406), Clopidogrel (337/1023). **Major bleeding**: Prasugrel (9/406), Clopidogrel (63/1023).	12 months
**2. Reduced-Dose (3.75 mg)**					
	Isshiki, T. (2014) # 58	742	**Prasugrel** (20-mg LD/3.75-mg daily) versus **Clopidogrel** (300-mg LD/75-mg daily).	**MACE**: Prasugrel (15/370), Clopidogrel (25/372). **Major bleeding**: Prasugrel (0/370), Clopidogrel (8/372).	24 - 48 weeks
	Saito, S. (2014) # 13	1,363	**Prasugrel** (20-mg LD/3.75-mg daily) versus **Clopidogrel** (300-mg LD/75-mg daily).	**MACE**: Prasugrel (74/685), Clopidogrel (84/678).	24 - 48 weeks

			**Major bleeding**: Prasugrel (13/685), Clopidogrel (15/678).	
Kimura, T. (2015) # 8	422	**Prasugrel** (20-mg LD/3.75- or 5-mg daily) versus **Clopidogrel** (300-mg LD/75-mg daily).	**MACE**: Prasugrel (6/141), Clopidogrel (8/140). **Major bleeding**: Prasugrel (0/141), Clopidogrel (2/140).	4 – 12 weeks
Ohya, M. (2018) # 40	1,167	**Prasugrel** (20-mg LD/3.75- or 2.5-mg daily) versus **Clopidogrel** (300-mg LD/75-mg daily).	**MACE**: Prasugrel (2/487), Clopidogrel (6/192). **Major bleeding**: Prasugrel (3/487), Clopidogrel (5/192).	12 months
Yasuda, S. (2019) # 59	3,069	**Prasugrel** (20-mg LD/3.75-mg daily) versus **Clopidogrel** (300-mg LD/75-mg daily).	**MACE**: Prasugrel (177/2607), Clopidogrel (46/462). **Major bleeding**: Prasugrel (99/2607), Clopidogrel (36/462).	12 months
Kitano, D. (2020) # 31	78	**Prasugrel** (20-mg LD/3.75-mg daily) versus **Clopidogrel** (300-mg LD/75-mg daily).	**MACE**: Prasugrel (5/37), Clopidogrel (4/38).	12 months
Kitagawa, K. (2020) # 60	654	**Prasugrel** (20-mg LD/3.75- or 2.5-mg daily) versus **Clopidogrel** (300-mg LD/50-mg daily).	**MACE**: Prasugrel (9/216), Clopidogrel (8/223). **Major bleeding**: Prasugrel (0/216), Clopidogrel (0/223).	48 weeks
Shibata, K. (2021) # 61	534	**Prasugrel** (20-mg LD/3.75-mg daily) versus **Clopidogrel** (300-mg LD/75-mg daily).	**MACE**: Prasugrel (17/204), Clopidogrel (35/330). **Major bleeding**: Prasugrel (4/204), Clopidogrel (3/330).	3 months
Kitazono, T. (2023) # 62	2,688	**Prasugrel** (20-mg LD/3.75-mg daily) versus **Clopidogrel** (300-mg LD/75- or 50-mg daily).	**MACE**: Prasugrel (45/1337), Clopidogrel (58/1351). **Major bleeding**: Prasugrel (2/1337), Clopidogrel (1/1351).	PRASTRO-I: 96–104 weeks, PRASTRO-II: 48 weeks, and PRASTRO-III: 24–48 weeks.

Kitazono, T. (2023) # 63	230	**Prasugrel** (20-mg LD/3.75-mg daily) versus **Clopidogrel** (300-mg LD/75-mg daily).	**MACE**: Prasugrel (8/118), Clopidogrel (8/112). **Major bleeding**: Prasugrel (1/118), Clopidogrel (0/112).	24– 48 weeks
Kuno, T. (2024) # 64	5,392	**Prasugrel** (20-mg LD/3.75-mg daily) versus **Clopidogrel** (300-mg LD/75-mg daily).	**Major bleeding**: Prasugrel (9/659), Clopidogrel (109/4733).	24 months
